# Sporadic Cerebral Cavernous Malformations: Report of Further Mutations of CCM Genes in 40 Italian Patients

**DOI:** 10.1155/2013/459253

**Published:** 2013-08-22

**Authors:** Rosalia D'Angelo, Concetta Alafaci, Concetta Scimone, Alessia Ruggeri, Francesco Maria Salpietro, Placido Bramanti, Francesco Tomasello, Antonina Sidoti

**Affiliations:** ^1^Department of Biomedical Sciences and Morphological and Functional Images, Division of Medical Biotechnologies and Preventive Medicine, University of Messina, Via C. Valeria 1, 98125 Messina, Italy; ^2^IRCCS Centro Neurolesi “Bonino-Pulejo”, Via Palermo SS. 113, Contrada Casazza, 98122 Messina, Italy; ^3^Department of Neurosciences, University of Messina, Via C. Valeria 1, 98125 Messina, Italy; ^4^Biology and Cellular Biotechnologies, Department of Animal Biology and Marine Ecology, University of Messina, Salita Sperone 31, 98166 S. Agata, Messina, Italy; ^5^Clinic of Neurosurgery, United Hospitals “Papardo-Piemonte”, Contrada Sperone, 98158 Messina, Italy

## Abstract

Cerebral cavernous malformations (CCMs) are vascular lesions characterized by abnormally enlarged capillary cavities, affecting the central nervous system. CCMs can occur sporadically or as a familial autosomal dominant condition with incomplete penetrance and variable clinical expression attributable to mutations in three different genes: *CCM1* (K-Rev interaction trapped 1 (KRIT1)), *CCM2* (MGC4607), and *CCM3* (PDCD10). CCMs occur as a single or multiple malformations that can lead to seizures, focal neurological deficits, hemorrhagic stroke, and headache. However, patients are frequently asymptomatic. In our previous mutation screening, performed in a cohort of 95 Italian patients, both sporadic and familial, we have identified several mutations in CCM genes, three of which in three distinct sporadic patients. In this study, representing further molecular screening of the three CCM genes, in a south Italian cohort of CCM patients enrolled by us in the last three years, we report the identification of other four new mutations in 40 sporadic patients with either single or multiple CCM.

## 1. Introduction

Cerebral cavernous malformations (CCMs; OMIM 116860) are a common disorder found in 0.1 to 0.5% of the population, representing ~10 to 20% of cerebral vascular lesions [1].

These are characterized by a cluster of thin-walled vascular sinusoids lined by a single layer of endothelium, lacking an intervening neural parenchyma or identifiable mature vessel-wall elements [2].

Most often, CCMs occur sporadically, and individuals develop generally only one malformation and do not carry a CCM gene germline mutation. 

In familial CCM, the disorder is dominantly inherited, and individuals often develop multiple malformations [3, 4].

However, some CCM patients who have multiple MRI lesions do not have any known clinically affected relative and therefore present as sporadic cases [5]. 

Patients with multiple lesions constitute more than 50% in the familial form of CCMs and 12–20% in sporadic forms [6, 7]. Cavernous malformation may present with symptoms which include focal neurologic signs, hemorrhagic strokes, seizures, and headaches [1]. However, symptomatic disease is considerably less common and can be diagnosed incidentally [6]. In MRI studies, CCMs typically appear in T2-weighted images as “popcorn-like” masses. They consist of a mixed signal intensity core usually accompanied by a hypointense hemosiderin rim.

Causative mutations have been identified in three genes: *CCM1 *(K-Rev interaction trapped 1 (KRIT1)), *CCM2 *(MGC4607), and *CCM3 *(PDCD10) genes [8, 9], whose products, Krit1, malcavernin, and PDCD10, respectively, are involved in a common functional pathway.

Krit1 and malcavernin interact through a krit1/malcavernin PTB domain interaction, analogous to the krit1/ICAP1 PTB interaction. The krit1/malcavernin//ICAP1 complex may function in the nucleus, whereby the malcavernin sequesters krit1 or the krit1/ICAP1 complex in the cytoplasm as part of a signalling complex for MAPK or integrin-mediated signal transduction events [10].

Furthermore, the *CCM2* gene product also interacts with the *CCM3 *gene product (PDCD10), and this interaction is increased in the presence of krit1 [11]. PDCD10 is also related to apoptosis, an essential process in arterial morphogenesis [12]. 

These data indicate that the genetic heterogeneity observed in familial CCM may reflect mutation of different molecular members of a coordinated signalling complex.

In our previous study, we screened the CCM genes in a cohort of 95 Italian patients, either sporadic or familial, as well as their at-risk relatives. Seventeen mutations, three of which in three distinct sporadic patients, were identified [8].

Here, we report further variants of CCM genes in another 40 Italian sporadic patients.

## 2. Materials and Methods

### 2.1. Patients

A total of 40 CCM patients were consecutively enrolled on the basis of neuroradiologic diagnosis of CCM by MRI.

Patients with single or multiple malformations, but without known clinically affected relatives, were classified as apparently sporadic.

Clinical and neuroimaging information on the number and localization of CCM lesions was collected through direct interview and review of medical records.

The cohort was composed of 23 women and 17 men from some provinces of Sicily and Calabria (southern Italy) with an average age at presentation of 46.0 years. 

12/40 patients presented with seizures, 9/40 with headache, and 7/40 with focal neurological deficits, while 8/40 presented with multiple symptoms; 4/40 patients were asymptomatic, and the CCM lesions were identified incidentally. Lesion localisations were supratentorial in 23/40, infratentorial in 16/40, and supra/subtentorial in 1/40. Only 5/40 patients had multiple CCMs.  Table 1 shows clinical and neuroradiological features of CCM sporadic patients. 

Written informed consent for clinical investigations and molecular analysis was obtained from all patients enrolled in this study.

### 2.2. Molecular Analyses

CCM genes mutation analysis was also performed on a control group comprising 100 unrelated, randomly selected, healthy individuals (53 female and 47 male, aged 20–79 years), from the same geographical areas of the patients.

### 2.3. DNA Extraction, Polymerase Chain Reaction, and Sequencing

Genomic DNA was extracted from peripheral blood using standard salting out procedures. Coding exons and intron-exon boundaries of *CCM1*, *CCM2*, and *CCM3* genes were screened using the pairs of primers designed according to the *CCM1*, *CCM2*, and *CCM3* published nucleotide sequences of GenBank (accession numbers NG_012964.1, NG_016295.1, and NG_008158.1, resp.). The sequence variation detected was described according to the recommendations by the Human Genome Variation Society, having the A of the ATG translation starting codon as +1 at the cDNA level.

### 2.4. *Multiplex* Ligation-Dependent Probe Amplification (Mlpa) Assay

To exclude the presence of large genomic deletions and duplications, not detectable by direct sequencing, MLPA was performed according to the manufacturer's instructions using two MLPA kits (SALSA MLPA Kits, P130 and P131 CCM, MRC-Holland). For visual inspection, peak heights were compared between the sample and controls to find any alteration in relative peak heights within the test sample. For the normalized peak-area calculations, each peak area was normalized by dividing the individual peak area by the total peak area of all peaks for that sample.

### 2.5. PCR-RFLP (Restriction Fragment Length Polymorphism) Analysis

To characterize the two variants identified in 3′UTR-*CCM1* gene, PCR-RFLP analysis with the specific restriction enzymes was used. 

We performed two separate digestions: one with Bse YI (for c.*132T>G mutation characterization) and the other with AciI (for c.*137delC characterization).

Normal PCR product of 478 bp has two BseYI restriction sites, and, after digestion, three fragments were detected of 287, 89, and 102 bp. Likewise, AciI cut into two restriction sites giving three fragments of 297, 92, and 89 bp. 

In both cases, the T>G substitution and C deletion lead to the loss of the recognition sites for BseYI and AciI, respectively. 

To establish whether the mutations were in cis- or transconfiguration, a double digestion (BseYI+AciI) was performed, since we were not able to carry out the genomic analysis on patient's parents.

Variants not reported in the SNP database (http://www.ensembl.org/Homo_sapiens/Gene/Sequence?g=ENSG00000001631;r=7:91828283-91875480) and with a frequency, in healthy control group, of less than 1%, were classified as “mutation.”

### 2.6. In Silico Analysis

With regard to mutation c.1280 A>G (p.D427A) in *CCM2 *gene, and in order to search for motifs able to bind splicing regulatory proteins created, destroyed, weakened, or strengthened by the mutation, analysis was performed using SpliceAid (http://www.introni.it/splicing.html) [13] and Rescue-ese (http://genes.mit.edu/burgelab/rescue-ese/) [14]. NetGene2 tool (http://www.cbs.dtu.dk/services/NetGene2/) was also used to evaluate 5′ and 3′ splice sites. 

Provean (http://provean.jcvi.org/index.php) and SIFT Human Protein (http://sift.jcvi.org/www/SIFT_enst_submit.html) were used to predict the possible effects of the mutation on protein.

Prediction on the effects of the mutation on the stability of the protein was carried out by Mupro (http://www.ics.uci.edu/~baldig/mutation.html).

Furthermore, considering the possibility that the two mutations detected in the 3′ untranslated region (UTR) of the *CCM1 *gene may have an effect on the regulation of *CCM1* gene expression, bioinformatics tools such as RegRNA (http://regrna.mbc.nctu.edu.tw/html/prediction.html) and FindTar3 Online Prediction (http://bio.sz.tsinghua.edu.cn/) were used.

## 3. Results and Discussion

Four novel mutations in heterozygous condition were identified in three distinct sporadic patients: three in *CCM1* gene and one in *CCM2*. 

**Figure 1:**
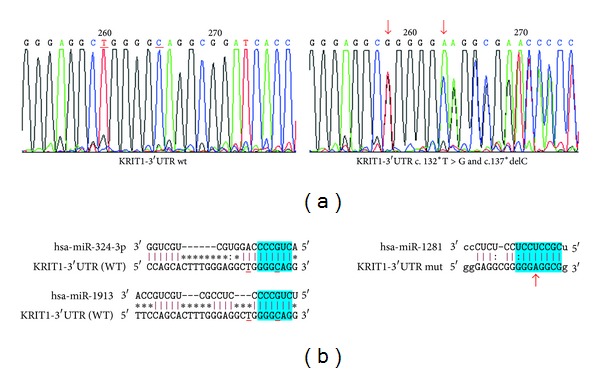
Identification of two mutations in 3′UTR of the *CCM1* gene in a sporadic CCM patient. (a) Left: partial wild-type sequence of 3′UTR of the *CCM1* gene; right: mutated sequence of 3′UTR of the *CCM1* gene showing the variants c.*132T>G and c.*137delC (arrows) in heterozygous condition. (b) Left: schematic representation of the sequence alignment of the hsa-miR-324-3p and hsa-miR-1913 with wild-type (WT) *CCM1* mRNA (sequence target); right: schematic representation of the sequence alignment of hsa-miR-1281 with mutated *CCM1* mRNA (sequence target). The “seed region” at the 5′ end of miRNA is shown in blue. While c.*132T>G is located 2 bp upstream of the predicted target sequence for has-miR-324-3p and hsa-miR-1913, the c.*137delC resides inside a sequence matching the seed of miRNAs. The c.*137 C deletion totally abolishes the interaction of hsa-miR-324-3p and hsa-miR-1913 with the sequence target. The two mutations together create a seed* site *in the 3′ UTR of mutated *CCM1* mRNA for the interaction with hsa-miR-1281.

In particular, in a sporadic patient with a single lesion, we have identified two mutations: c.*132T>G and c.*137delC, in the 3′ untranslated region of the *CCM1* gene, both in heterozygous condition (Figure 1(a)).

The mutations lie in exon 19 of *CCM1* gene, respectively, 132 and 137 bp after the TGA translation termination codon.

BseYI+AciI digestion shows a restriction pattern in which the presence of 478 bp DNA fragment, corresponding to wild-type allele, allowed us to establish that the two variants were in cisconfiguration.

To assess whether the mutations in 3′UTR-*CCM1 *gene may have an effect on the regulation of *CCM1* gene expression, as it fell within the target sequence for a specific miRNA, in silico analyses were undertaken.

RegRNA and FindTar3 Online Prediction indicate that the mutations are within the sequence target for hsa-miR-324-3p and hsa-miR-1913. In particular, while c.*132T>G resides 2 bp upstream of the sequence matching the seed of hsa-miR-324-3p and hsa-miR-1913, the c*137delC resides inside a sequence matching the seed of two miRNAs. The c.*137delC changes the predicted miRNA site from 3′GGGCAG5′ to 3′GGGAG5′, thus introducing a mismatch that completely abolishes the hsa-miR-324-3p and the hsa-miR-1913 interactions. The two mutations together create a sequence target in the 3′UTR of mutated *CCM1 *mRNA for the interaction with hsa-miR-1281 (Figure 1(b)).

Little is known about these hsa-miRNAs and their expression in human brain microvascular endothelial cells. With reagrd to hsa-miR-324-3p, its targets include mRNAs encoding proteins along much or all of the Wnt signalling pathway and Delta-Notch signalling [15], both involved in tumor associated angiogenesis.

Is also known that just Krit1 is a pivotal inhibitor of angiogenesis by Krit1–Rap1 interaction and Delta-Notch signalling activation [16, 17]. 

**Figure 2:**
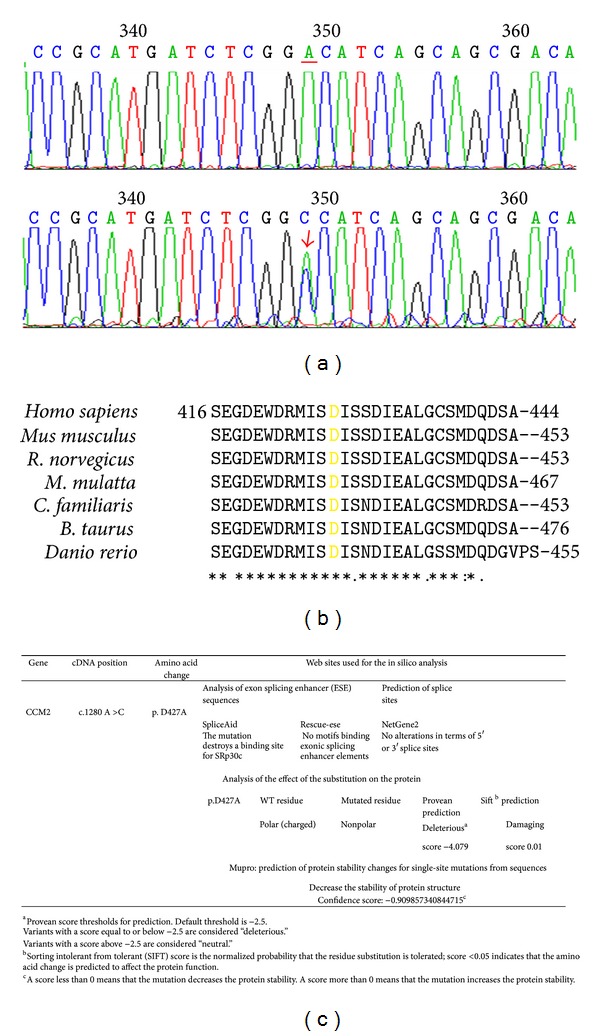
Identification of a mutation in exon 10 of *CCM2* gene in a sporadic CCM patient. (a) At the top: partial wild-type sequence of exon 10 of *CCM2* gene; at the bottom: partial mutated sequence of exon 10 of *CCM2* gene showing the mutation c.1280 A>G (arrow) in heterozygous condition. (b) The D427 residue, highlighted in yellow in the figure, is highly conserved among species. (c) Web informatics tools used for the in silico analysis and prediction results.

In a patient with multiple lesions, we found a missense mutation in exon 10, c.1280 A>G, (p.D427A), a change that did not obviously disrupt the open reading frame and is not near a splice junction (Figure 2(c)). The mutation lies in C-terminus (Karet domain) of the malcavernin protein, a domain required, together with PTB domain, for TrkA-dependent cell death [18]. The newly identified mutation affected the D427 residue, which is highly conserved among species (Figure 2(b)), and considering that this mutation is not found in other species, it is likely that aspartic acid is very important for intramolecular interactions for folding, conformation of malcavernin protein. 

To assess the effect of the D427A substitution on protein structure and function, we have applied two freely available web-based services, Provean and SIFT Human Protein. 

Both programs were developed to predict whether a specific amino acid substitution is deleterious for protein function.

The programs rely on structural information obtained with crystallographic or NMR methods, on other functional and structural characterizations and on primary sequence comparison, and are trained on a number of proteins and mutants having known mutation-phenotype relationships. If sufficient homology and structural data are available for a specific protein, both programs predict neutral or deleterious substitutions providing a score of significance for each prediction. Both programs predict a deleterious effect of the amino acidic substitution on protein structure and function. The results of these analyses are summarized in Figure 2(c).

Regarding splicing prediction analysis, SpliceAid tool highlights that the mutation destroys a binding site for SRp30c, a factor acting as an exonic splicing enhancer protein. 

 Rescue-ese, instead, does not show motifs that bind exonic splicing enhancer elements, in either the original or the mutated sequence. 

According to NetGene2 tool there are no alterations in terms of 5′ or 3′ splice sites (Figure 2(c)).

Prediction of protein stability changes for single-site mutations from sequences (Mupro) shows that the amino acidic substitution decreases the stability of protein structure (Figure 2(c)).

**Figure 3:**
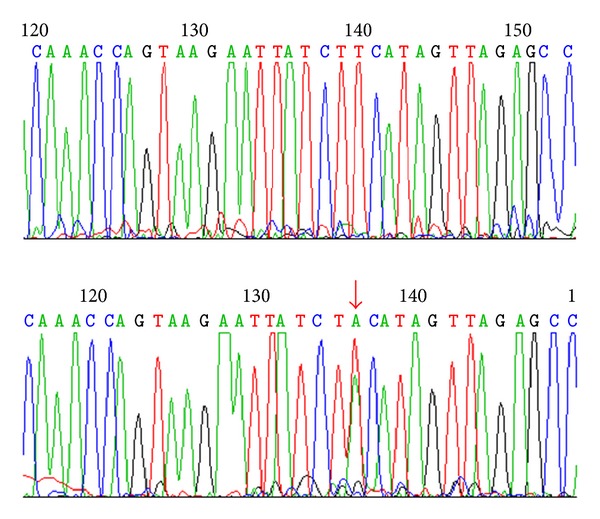
Identification of an intronic mutation in *CCM1* gene, c.1254+14 T>A (IVS12+14 T>A).

Finally, we have detected an intronic mutation in *CCM1* gene, c.1254+14 T>A (IVS12+14 T>A) in a third patient with a single lesion (Figure 3). 

NetGene2 v. 2.4 prediction tool did not indicate alterations in either donor or acceptor splice sites. 

In addition to these mutations, twelve variants in three CCM genes were identified, which proved to be the known polymorphisms, based on the fact that they have been observed in both patients and controls. All variants were reported elsewhere in databases. 

In particular, in* CCM1* gene three polymorphisms were identified: two were intronic, IVS 10+63G>C (rs2027950) and IVS15-90 G>C (rs58251940), while one was a silent substitution in exon 17, c.A1980G (rs11542682) [19]. 

In *CCM2 *gene, seven variants were identified. Among these, four were located in coding region: exon 2 c.157 G>A (rs2107732), exon 4 c.358 G>A (rs11552377), exon 8 c.915 G>A (rs2289367), and c.881 G>A (rs144648280); three were intronic: IVS2-36 A>G (rs2304689), IVS4+127 C>T (rs73107990), and IVS8+119 C>T (rs2289369). 

In agreement with our previous case-control study, these variants were detected in patients who were negative for CCM mutations.

Further on in this study, we had found that IVS2-36 A>G and c.915 G>A polymorphisms were associated with a significant increase in risk for CCM, while the c.358 G>A and c.915 G>A polymorphisms predisposed the patients to a higher occurrence of a “potentially disabling” symptomatology (such as headache) rather than to a “possibly life-threatening” symptomatology (such as epilepsy, cerebral haemorrhage, and other symptoms) [20].

In *CCM3*, we have detected two variants: c.150 G>A (rs116154329) in exon 2 and IVS3+53 C>T (rs200180968) (Table 2).

The present study represents further molecular screening of the three genes in a south Italian cohort of CCM patients enrolled by us in the last three years of research study. All 40 patients, 36 symptomatic and 4 asymptomatic, were screened for mutations in *CCM1*, *CCM2*, and *CCM3* genes by DNA direct sequencing. The screening led to identification of four new mutations. 

In particular, two of these mutations were identified in a symptomatic patient with a single lesion. 

The mutations, c.*132T>G and c.*137delC, fell in the 3′ untranslated region of the *CCM1* gene, within the sequence target for hsa-miR-324-3p and hsa-miR-1913. In silico analyses showed that c.*137delC introduced a mismatch that completely abolished the hsa-miR-324-3p and hsa-miR-1913 interactions. The two mutations together created a sequence target in the 3′ UTR of mutated *CCM1* mRNA for interaction with hsa-miR-1281.

These changes in the interactions between miRNAs and their sequence target may have effects on *CCM1* gene expression. Unfortunately, we do not have endothelial cells from CCM lesions that can be used versus control endothelial cells to validate the prediction that these mutations lead to the abolition of hsa-miR-324-3p and hsa-miR-1913 interactions, since the patient harboring these mutations has not been subjected to surgery. 

Cisconfiguration of two mutations is an important element to consider. 

It is known, in fact, that two mutations in cisconfiguration and then on the same allele left the second allele intact, while if in trans and then on opposite alleles no functional form of the gene is present.

This is particularly significant when one considers that CCMs, including sporadic forms, develop in response to alterations in both copies of a gene, according to the two-hit model.

In sporadic cases, two random somatic mutations occur in the same gene within the same cell. In familial cases, individuals inherit one germline mutation, and, thus, only one random somatic second-hit mutation in the same gene is necessary for pathogenesis [21].

The cisconfiguration could determine the possibility that an individual carrying the two mutations can transmit the intact allele to offspring.

An intronic mutation in *CCM1* gene, c.1254+14 T>A (IVS12+14 T>A), was identified in a symptomatic patient with a single lesion, while a missense mutation in *CCM2* exon 10, c.1280 A>G, (p.D427A), was identified in a patient with multiple lesions. This mutation fell in the C-terminus (Karet domain) of the malcavernin protein and caused the deleterious substitution of the aspartic acid to alanine. 

No intragenic deletions or duplications by MLPA were identified in patients negative to direct exon sequencing.

In addition to these mutations, twelve variants in three CCM genes were identified, which proved to be the known polymorphisms.

## 4. Conclusions

Genomic analysis of 40 sporadic patients shows that only 7.5% (3/40) of patients harbour CCM mutations. Two out of four mutations identified were detected in the same patient with a single lesion: one was a point mutation and the other was a single nucleotide deletion, both in* CCM1 *gene. The remaining mutations were point mutations, identified in *CCM1* and *CCM2* genes in two patients with a single lesion. 

*CCM1* and *CCM2* mutations were found in 5 (2/40) and 2.5% (1/40) of our sporadic patients, while no mutations were identified in *CCM3*.

Thus, our mutation detection rates for the three genes were in disagreement with the results obtained from larger French [22, 23], Swiss [24], and Japanese [25] cohorts. No *CCM3* mutations were identified in another Italian cohort where, however, *CCM1* mutation rate was equal to 1.3% [8].

92.5% of our patients do not present mutations in one of the CCM genes. This value is significantly higher than in some of the previous series, suggesting the existence of mutations in unidentified CCM genes, other than *CCM1*, *CCM2*, and *CCM3* [26].

Unlike Verlaan et al. 2004 [27] who did not find *CCM2* mutations in German sporadic patients with multiple lesions, in our cohort, 20% of the patients with multiple lesions (1/5) have a CCM mutation and to be precise in *CCM2* gene. No *CCM1* mutations were found in these patients, in disagreement with what has already been reported in the literature [24, 28].

From literature, it is known that the mutation detection rate was lower in sporadic cases with multiple lesions, ranging from 45% to 67% [22, 29, 30].

Since sporadic cases are mainly characterized by the presence of a single lesion, it is possible that sporadic cases with multiple lesions have inherited their mutation from one of their asymptomatic parents because of incomplete penetrance or have a de novo mutation. 

The four mutations identified extend the genetic mutation spectrum of CCMs and can be helpful to assess testing and counselling of CCM patients and their families. Data are important for a better counselling and for disease management.

## Figures and Tables

**Table 1:** Clinical and neuroradiological features of CCM sporadic patients.

Feature	All subjects	Males	Females
	40	*N* = 17	*N* = 23
Symptomatic subjects	*N* = 36	*N* = 17	*N* = 19

Age of patients (yr)			
Mean ± SD	45.6 ± 16.3	51.35 ± 16.04	40.5 ± 12.8
Range	2–77	18–77	2–60
Age of onset (yr)			
Mean ± SD	41.0 ± 20.0	51.3 ± 16.0	40.5 ± 12.8
Range	1–71	18–77	2–60
			
Number of CCM lesions			
Single	31	13	18
Multiple	5	4	1
Lesion seat	23 supratentorial
	12 subtentorial
	1 supra/subtentorial
Extracerebral	0		
Types of first clinical manifestations			
Cerebral haemorrhages			
Cerebral haemorrhages + headache (1)	4		
Cerebral haemorrhages + focal neurological deficits (3)			
Epilepsy			
Epilepsy (12)	14		
Epilepsy + focal neurological deficits (2)			
Focal neurological deficits			
Focal neurological deficits (7)	9		
Focal neurological deficits + headaches (2)			
Headaches	**9**		

Asymptomatic subjects	N = 4	N = 0	N = 4

Age of patients (yr)			
Mean ± SD	48.2 ± 13.0		
Range	36–64		
Number of CCM lesions			
Single	4		
Multiple			
Lesion seat	Subtentorial		
Extracerebral	0		

**Table 2:** *CCM1*, *CCM2*, and *CCM3* polymorphisms.

	Exon	Sequence variation	Coding change	SNP reference
*CCM1 *	10	c.989+63 C>G	Intronic	rs2027950
		IVS10+63 C>G		
	16	c.1731−90 G>C	Intronic	rs58251940
		IVS15−90 G>C		
	17	c.1980 A>G	p.V660V	rs11542682

*CCM2 *	2	c.157 G>A	p.Val53Ile	rs2107732
	3	c.205−36 A>G	Intronic	rs2304689
		IVS2−36 A>G		
	4	c.358 G>A	p.Val120Ile	rs11552377
	4	c.472+127 C>T	Intronic	rs73107990
		IVS4+127 C>T		
	8	c.881 G>A	p.S294N	rs144648280
	8	c.915 G>A	p.Thr305Thr	rs2289367
	8	c.915+119 C>T	Intronic	rs2289369
		IVS8+119 C>T		

*CCM3 *	2	c.150 G>A	p.K50K	rs116154329
	3	c.268+53 C>T	Intronic	rs200180968
		IVS3+53 C>T		

## Abbreviations

MRI: Magnetic resonance imaging
PDCD10: Programmed cell death 10
PTB domain: Phosphotyrosine-binding domain
ICAP-1*α*: Integrin cytoplasmic domain-associated protein-1*α*
MAPK: Mitogen-activated protein kinase
SNP: Single nucleotide polymorphism
TrKA: Receptor tyrosine kinases A.
